# Advances in Plant Autophagy

**DOI:** 10.3390/cells10010194

**Published:** 2021-01-19

**Authors:** Agnieszka Sirko, Céline Masclaux-Daubresse

**Affiliations:** 1 Institute of Biochemistry and Biophysics, Polish Academy of Sciences, ul. Pawinskiego 5A, 02-106 Warsaw, Poland; 2 Institut Jean-Pierre Bourgin, INRAE, AgroParisTech, Université Paris-Saclay, 78000 Versailles, France

Ubiquitin–proteasome and lysosome–autophagy are the two main cellular degradation systems controlling cellular homeostasis in eukaryotes. The autophagy pathway started attracting particular attention only two decades ago, after the ATG genes were discovered in yeast, and their counterparts in higher eukaryotes. Since then, a tremendous amount of autophagy-related knowledge was gained leading to deciphering its molecular mechanisms and their regulations. Notably, the significance of autophagy in the pathophysiology of human disease and aging was demonstrated [1]. These findings stimulated and accelerated the work progress on the possible roles of autophagy in different organisms. Although the autophagy pathway is evolutionarily conserved, substantial differences in lifestyle, overall structure and morphology impose the existence of numerous kingdom-specific features. The work on the function of autophagy in plants is lagging behind the studies performed on mammals, which are motivated by the potent therapeutic function of autophagy in metabolic, cardiovascular, neurodegenerative, inflammatory and other diseases. In addition to the basic research dedicated to understanding the molecular mechanisms of autophagy [2,3], the work in plants is also motivated by potential biotechnological applications related to the role of autophagy in stress response [4] and plant performances in agriculture [5].

This Special Issue of *Cells* includes nine articles, providing an insight into the current progress on autophagy-related research in plants. Here, the significance of autophagy in nutrient management is reported in several articles. In a review article, Chen et al. [6] summarized the role of autophagy in the recycling and remobilization of nitrogen and other nutrients as iron, manganese and zinc during plant senescence. This highlights the links between autophagy, inorganic phosphate and carbon assimilation. In addition, the cross-talk of autophagy and senescence-related cysteine proteases is discussed, and the accumulation of some specific proteases in autophagy-deficient mutants suggest that these enzymes provide potential compensatory mechanisms and alternative nitrogen remobilization pathways to autophagy in low nitrogen conditions. The major conclusion of the work by Bedu at al. [7] is that the basal autophagy activity might be a part of the integral response of the nitrogen metabolism to nitrate availability; the mRNA steady state levels of ATG genes and of nitrogen assimilation-related enzymes are strongly correlated regardless of the nitrogen nutrition status. The study by Lornac et al. [8] used N and S isotopes to show that autophagy is also involved in sulphur remobilization from rosette leaves to seeds. However, depending on the sulphate availability in the soil, the nature of the sulphur-mobile molecules mobilized through the autophagy-dependent remobilization process is different. In line with the role of autophagy in nutrient remobilization, the study by Lopez-Vidal et al. [9] confirms the role of autophagy in metabolic changes occurring during pepper fruit ripening and reveals its implication in the recycling of organelles in this crop. The work by Tarnowski et al. [10] focuses on the role of the selective autophagy cargo receptor NBR1. The authors postulated that NBR1 can fine-tune plant response to sulphur deficit, by controlling the selective degradation of multiple targets in sulphur-deficient conditions. Two examples of such potential targets are provided, ribosomal protein S6 (RPS6) recognized by NBR1 in an ubiquitin-independent way and the kinase of RPS6 recognized by C-terminal UBA (ubiquitin associated) domain of NBR1. Interaction with these (and possibly other) targets might influence ribosome activities and/or biogenesis during sulphur-deficit and possibly other stresses. It is worth highlighting that a list of the proteins targeted at NBR1-mediated autophagy in different stresses has been recently reviewed [11].

The role of autophagy in ribosome turnover (ribophagy) is the subject of the interesting review by Kazibwe et al. [12]. The regulation of ribosome quality and the number is essential for cell homeostasis, to maintain proper translation, and to cope with environmental stresses. Authors discuss the autophagy-dependent degradation of ribosomal RNA and ribosomal proteins in yeast and animals and summarize evidence for the existence of ribophagy in plants.

Autophagy, as a part of the vesicular transport system, shares some components with other vesicular trafficking machineries, such as endo- and exo-cytic pathways. Their crosstalk must be coordinated during plant responses to abiotic and biotic stresses. The links between autophagosomes (double-membrane vesicles) as part of the autophagy pathway, and multivesicular bodies (single-membrane vesicles) as part of the endocytic system, is thoroughly discussed by Wang et al. [13]. The review focuses on the coordinated action of these two pathways during the hormone-mediated response of plants to biotic and abiotic stresses. Extensive interactions in the regulation and function of both types of vesicles in yeast and animals are also discussed.

The structural and mechanistic studies on the protein machinery involved in autophagy initiation and autophagosome biogenesis, are summarized in the review by Lai et al. [14]. Structural data mainly obtained by X-ray crystallography, are mostly only available for the yeast and mammalian proteins and complexes. Notably, the structure of the trimeric Arabidopsis ATG9 protein that was recently determined by cryoelectron microscopy, is the only available structure of ATG9 discovered to date. This represents a milestone in the field of plant autophagy that will facilitate further works in yeast, animal and plants on the function of this transmembrane protein in the biogenesis of autophagosomes.

All the articles cited above focused on macroautophagy, which is the most well studied type of autophagy in plant and relies on autophagosome biogenesis. The review by Sienko et al. [15] introduces plant microautophagy that consists of the direct uptake of cargoes into the vacuole by the invagination of the vacuolar/lysosomal membrane. Authors discuss evidence for microautophagy in yeast, animals and plants, and consider possible techniques, based on the methods and experience of other organisms, to study this process in plants.

Overall, this Special Issue provides important information on the recent advances in autophagy in plants, and the potential future directions of autophagy-related studies.
